# A nutrition and lifestyle-focused shared medical appointment in a resource-challenged community setting: a mixed-methods study

**DOI:** 10.1186/s12889-022-12833-6

**Published:** 2022-03-07

**Authors:** Nazleen Bharmal, Michelle Beidelschies, Marilyn Alejandro-Rodriguez, Kayla Alejandro, Ning Guo, Tawny Jones, Elizabeth Bradley

**Affiliations:** 1 Community Health & Partnerships, Cleveland Clinic Community Care, Cleveland Clinic, Ohio Cleveland, USA; 2 Center for Functional Medicine, Cleveland Clinic, Cleveland, Ohio USA; 3 Quantitative Health Sciences, Cleveland Clinic, Cleveland, Ohio USA

**Keywords:** Shared medical appointments, Lifestyle intervention, Dietary intervention, Low-income participants, Disparities

## Abstract

**Background:**

In order to address disparities in preventable chronic diseases, we adapted a nutrition and lifestyle-focused shared medical appointment (SMA) program to be delivered in an underserved community setting. The objective was to evaluate a community-based nutrition and lifestyle-focused SMA as it relates to acceptability and health and behavior-related outcomes.

**Methods:**

A mixed-methods study was performed to evaluate pre-post changes in wellness indices, biometrics, self-efficacy, and trust in medical researchers as part of a community-based SMA. To understand program acceptability including barriers and facilitators for implementation and scalability, we conducted two participant focus groups and five stakeholder interviews and used content analysis to determine major themes.

**Results:**

Fifteen participants attended 10 weekly sessions. The majority were older adult, African American women. There were pre-post improvements in mean [SD] systolic (-10.5 [7.7] mmHg, *p* = 0.0001) and diastolic (-4.7 [6.7] mmHg, *p* = 0.17) blood pressures and weight (-5.7 [6.3] pounds, *p* = 0.003) at 3 months though these were not significant at 6 months. More individuals reported improvements in health status, daily fruit and vegetable intake, and sleep than at baseline. There were no significant pre-post changes in other wellness indices, self-efficacy, trust in medical researchers, hemoglobin A1c, insulin, or LDL cholesterol. Participants discussed positive health changes as a result of the SMA program, program preferences, and facilitators and barriers to continuing program recommendations in focus groups. SMA implementation was facilitated by clinical staff who adjusted content to a low health literacy group and partnership with a trusted community partner. Sustainability barriers include heavy personnel time and in-kind resources to deliver the program.

**Conclusions:**

Nutrition and lifestyle-focused SMAs in a resource-challenged community setting may be an acceptable intervention for underserved patients.

## Background

African Americans and/or individuals of lower socioeconomic status have a disproportionate burden of nutrition and lifestyle-related chronic diseases, such as obesity, high blood pressure, and diabetes [1, 2]. This difference is partially due to neighborhood factors, such as living in resource-challenged environments with few outlets for affordable nutritious food, limited safe places for physical activity, and inadequate access to healthcare facilities [3–5].

Shared medical appointments (SMAs), or group visits, are a potential health-care delivery solution for the management of various chronic conditions [6–9]. Briefly, SMAs involve multiple visits which incorporate a multi-disciplinary team of caregivers to deliver medical care and/or education to groups of patients with the same medical condition. SMAs are cost-effective solutions to healthcare delivery designed to improve health outcomes and increase access to care [8]. They provide patients with an opportunity to support each other which is an essential part of the model of care [7].

A narrative review found SMAs for low-income and underserved populations, including racial/ethnic minorities, to be effective in diabetic preventive care [9]. However, most studies were performed in clinic- or hospital-based settings which are associated with higher rates of distrust in African-Americans with lower socioeconomic status [10]. Such settings may impede engagement and participation and, ultimately, impact health outcomes. Therefore, delivery of SMAs directly in community-based settings with neighborhood disadvantage may be more acceptable and result in improved health outcomes; however, few studies have examined this. Therefore, the objective of the current study was to evaluate a community-based nutrition and lifestyle-focused SMA to assess acceptability as well as health and behavior-related outcomes.

## Methods

### Community-based SMA program

In partnership with healthcare system-based community engagement staff, the Center for Functional Medicine designed a community-based SMA program for people living in a resource-challenged Cleveland neighborhood. The community-based SMA program was offered as a community benefit and adapted from the clinic-based SMA program [6]. The clinic-based SMA from the Center for Functional Medicine is a 10-week program that provides education related to nutrition and lifestyle and provides behavioral health recommendations [6]. Providers deliver education and care in a shared environment plus a brief, individual medical assessment. Health coaches provide education related to exercise and movement, sleep, stress reduction and tools to support lasting behavioral change. Dietitians focus on the use of food as medicine, and support participants in the implementation of a food plan that encourages consumption of whole, unprocessed foods. By the end, participants are empowered to make positive decisions regarding food and become advocates within their homes and communities. For the community-based adaption, the SMA focused on weight management utilizing a cardiometabolic food plan similar to the Mediterranean Diet [11]. It consisted of weekly, in-person group sessions with four sessions led by a clinical practitioner (PA-C) and health coach, and six sessions led by a registered dietitian. Each session lasted for 1–2 h.

The community-based SMA sessions delivered nutrition and lifestyle-related education, provided participants with educational tools, and fostered open discussion. Participants were provided customized shopping lists for cooking and menu options for eating out. A cooking demonstration session helped participants appreciate how to prepare certain foods. As part of the SMA, community participants received in-kind laboratory testing, dietary supplementation (Pure Lean Pure Pack and Vitamin D (Pure Encapsulations, LLC)), and weekly food delivery (Freshly, Inc.) for themselves and three additional members of their household.

### Study design and population

The study design was a mixed-methods, pre-post survey of a community-based SMA. Prior to the start of the SMA, participants self-completed a written survey and provided baseline biometric measurements and laboratory testing. At 3 months (after completion of the SMA), participants self-completed a written post-SMA survey and provided post-SMA biometrics and laboratory testing. We also conducted a focus group discussion among the SMA participants to discuss their experiences with the community-based SMA program at 3 months. We repeated biometric testing at 6 months (e.g., 3 months after completion of the SMA) and conducted a second focus group with the SMA participants to explore retention of knowledge and habits.

The community-based SMA program was held at the Langston Hughes Community Health and Education Center in the Fairfax neighborhood of Cleveland, Ohio. SMA participants lived in the Fairfax neighborhood. Fairfax is home to over 6,000 people with 94% identifying as Black or African American and 62% having a high school diploma or less educational attainment [12]. The majority of households are in or near poverty which is a result of years of redlining, disinvestment, and population decline [12]. The neighborhood population suffers from a number of health disparities compounded by social determinants of health, such as higher rates of heart disease, cancer, diabetes, and kidney disease than the surrounding areas.

Study participants had to be ≥ 18 years old, attend at least one community-based SMA session, and have previously participated in prior health education activities at the Langston Hughes Center. Exclusion criteria included inability to complete paper surveys and/or inability to sit through a 60-minute focus group discussion. All participants provided written consent to participate in the research study.

We also conducted five programmatic stakeholder interviews to discuss the implementation of the community-based SMA program. Programmatic stakeholders were those who provided administrative support and/or delivered the program.

### Survey and biometrics

SMA participants completed a written survey at baseline and 3 months (post-SMA). Survey items included demographic information and questions on wellness indices [13], food security [14], self-efficacy [15], and trust in medical research [16, 17] that were adapted from validated instruments. Wellness indices were adapted from the Behavioral Risk Factor Surveillance System Survey and included self-reported health status, fruit/vegetable intake, physical activity, sleep duration, stress levels, alcohol and tobacco use [13]. Survey responses on food security, self-efficacy, and trust in medical research were on a 5-point Likert scale from strongly disagree to strongly agree. Food security was assessed by two validated items: (1) Within the past 30 days, we worried whether food would run out before we got money to buy more and (2) Within the past 30 days, the food we bought just didn’t last and we didn’t have money to get more [14]. General self-efficacy was assessed by 8 items with possible scores ranging from 8 to 40; higher scores represent greater perceived self-efficacy [15]. Trust in medical researchers was assessed by 5 items with scores ranging from 5 to 25; higher scores represent greater trust [17]. Participants received $10 for each survey completion.

Weight and blood pressure were assessed at baseline, 3 months, and 6 months. Laboratory testing for hemoglobin A1c (HbA1c), fasting insulin and low-density lipoprotein (LDL) cholesterol levels was evaluated at baseline and 3 months.

### Focus groups and stakeholder interviews

Two, 60-minute focus groups were conducted with SMA participants and facilitated by the principal investigator (PI). The first focus group was conducted at 3 months (i.e., one week after the completion of the community-based SMA program). In order to assess program acceptability, participants were asked to discuss their experiences in the program. The second focus group was conducted at 6 months (i.e., three months following the completion of the community-based SMA program). Participants were asked to discuss factors influencing the maintenance of positive health-related behaviors. Study participants received a $30 incentive for participation in each focus group.

The PI conducted one-on-one, 60-minute stakeholder interviews using a semi-structure survey. Interviews aimed to determine general and site-specific factors associated with greater effectiveness, and to assess the acceptability and sustainability of the community-based SMA program. Interview questions were guided by standard implementation analyses [18] and asked the following: What are the organizational resources to carry out the intervention? What are the staff experience and capacity to carry out the intervention? What are the potential barriers and facilitators to implementing the intervention? What potential modification to the intervention would need to be made to maximize implementation?

Focus groups and stakeholder interviews were recorded by audio-tape and through researchers’ notes.

### Statistical analysis

We described baseline characteristics for community-based SMA participants. Pre- and post-SMA biometrics and self-reported survey items were compared using paired t-test, McNemar’s test, or Fisher’s exact test. We compared participants’ pre- and post-SMA mean scores for general self-efficacy and trust in medical researchers. Statistical analyses were conducted using SAS version 9.4 (SAS Institute, Inc. Cary, NC). Statistical significance was established at *p* < 0.05. Results were shared in aggregate with the community partners and study participants.

Transcribed audio-taped sessions of focus groups were read by at least three research-eligible staff who were present during the focus group discussions. Staff first individually read transcripts and then discussed as a group using content analysis methods to identify and discuss main themes (kappa = 0.70 for inter-rater reliability). Focus group themes were shared with SMA participants to confirm accuracy. Stakeholder interviews were read by the PI and analyzed for main themes. Results were shared with stakeholders to confirm accuracy of themes associated with the implementation and sustainability of the community-based SMA program.

## Results

### Quantitative outcomes

Fifteen people from the Fairfax neighborhood participated in the community-based SMA program; all chose to participate in the research study. The majority of participants were older adult, African American women (Table 1). Half the participants reported living with high blood pressure, one-third with diabetes, and one-fifth with sleep apnea. Most reported having health insurance and a primary care doctor. While almost all the participants reported feeling confident in meal preparation with vegetables, there were varied responses to healthful food availability and food security. All participants attended the 10 weekly sessions of the SMA program, which was facilitated by the community engagement staff providing phone call reminders. One participant died about 2 months after program completion.

**Table 1:** Characteristics of community-based SMA program participants (*n* = 15)

	n (%)
**Sex**	
Female	13 (86.7)
Male	2 (13.3)
**Age**	
18–40 years	0 (0.0)
41–64 years	7 (46.7)
65–80 years old	8 (53.3)
**Race/Ethnicity** ^a^	
African-American/Black	13 (86.7)
Caucasian/White	1 (6.7)
**Educational attainment**	
<High school graduate	0 (0.0)
HS diploma, GED	5 (33.3)
Vocational school or some college	5 (33.3)
College or higher	5 (33.3)
**Chronic conditions**	
High Blood Pressure	8 (53.3)
Diabetes	5 (33.3)
Sleep Apnea	3 (0.2)
**Health insurance** ^a^	
Yes	14 (93.3)
No	0 (0.0)
**Primary Care Provider** ^a^	
Yes	14 (93.3)
No	0 (0.0)
**Feel confident in knowing how to prepare fresh vegetables for meals**	
Strongly agree/agree	14 (93.3)
Strongly disagree/disagree	0
Neutral	1 (6.7)
**Easy to buy fresh fruits and vegetables in my neighborhood**	
Strongly agree/agree	8 (53.3)
Strongly disagree/disagree	1 (6.7)
Neutral	6 (40.0)
**Within the past 30 days, we worried whether our food would run out before we got money to buy more**	
Strongly agree/agree	3 (20.0)
Strongly disagree/disagree	4 (26.7)
Neutral	8 (53.3)
**Within the past 30 days, the food we bought just didn’t last and we didn’t have money to get more**	
Strongly agree/agree	2 (13.3)
Strongly disagree/disagree	10 (66.7)
Neutral	3 (20.0)

^a^Missing = 1

Table 2 reports change in self-reported outcomes at 3 months. Four participants moved from fair or poor health status to good health status. More participants reported increased food and vegetable intake and sleep following completion of the program; however, these did not reach statistical significance. There was no change in physical activity or stress level. None of the participants reported heavy alcohol use or smoking. There was no change in perceived self-efficacy (mean score pre-SMA 31.7 and post-SMA 32.8 out of 40) or trust in medical researchers (pre-SMA 20, post-SMA 20.4 out of 25) with relatively high baseline scores for both scales.

**Table 2:** Change in self-reported wellness indices for community–based SMA program participants

		No. (%)		
	**N**	**Baseline**	**3 months**	***P*** **-value** ^†^
**Health Status** Excellent or very good Good Fair or poor	15	2 (13.3) 9 (60.0) 4 (26.7)	2 (13.3) 13 (86.7) 0 (0)	0.19
**Fruits and Vegetable Intake** ^a^ 5 or more servings/day	12	6 (50.0)	10 (83.3)	0.22
**Physical activity** ^a^ More than 150 min/week	14	14 (100.0)	13 (86.7)	n/a
**Sleep** 7 or more hours/night	15	6 (40.0)	9 (60.0)	0.25
**Stress** Rarely or Sometimes	15	12 (80.0)	13 (86.7)	1.00
**Alcohol consumption** Less than 7 drinks/week (women) or 14 drinks/week (men)	15	15 (100.0)	15 (100.0)	n/a
**Tobacco Use** No	15	15 (100.0)	15 (100.0)	n/a

^†^*P*-Value for the change from baseline to 3 months

^a^Data not available for all subjects. Missing values: Fruits and Vegetable Intake = 3; Physical Activity = 1

Table 3 reports change in biometric outcomes at 3 months. Participants experienced a reduction in their mean [SD] weight (-5.7 [6.3] pounds, *p* = 0.0034), and systolic (-10.5 [7.7] mmHg, *p* = 0.0001) and diastolic (-4.7 [6.7] mmHg, *p* = 0.017) blood pressures. Weight and blood pressure were available at 6 months for a subset of community-SMA participants and while there were improvements, they did not reach statistical significance. There was no significant change in mean HbA1c, total insulin or LDL cholesterol levels at 3 months.

**Table 3:** Change in biometrics for community-based SMA program participants

	Mean (SD)				Mean (SD)			
	**Baseline**	**3 months**	**Change** ^a^	***P*** **-Value**	**Baseline**	**6 months**	**Changea**	***P*** **-Value**
**N**	15	15			8	8		
**Weight, lbs.**	225.9 (59.7)	220.1 (56.6)	-5.7 (6.3)	**0.003**	195.9 (49.3)	193.1 (50.9)	-2.8 (7.2)	0.30
**Systolic Blood Pressure, mm/Hg**	132.2 (9.3)	121.7 (8.0)	-10.5 (7.7)	**0.0001**	132.6 (9.3)	126.9 (8.4)	-5.8 (10.0)	0.15
**Diastolic Blood Pressure, mm/Hg**	65.3 (8.9)	60.6 (10.6)	-4.7 (6.7)	**0.017**	62.9 (7.3)	64.1 (9.3)	1.3 (7.6)	0.66
**HbA1c, %**	6.1 (0.50)	6.2 (0.56)	0.11 (0.34)	0.05	n/a	n/a	n/a	n/a
**Insulin, mU/L**	13.2 (8.1)	11.6 (8.0)	-1.6 (3.7)	0.11	n/a	n/a	n/a	n/a
**LDL, Cholesterol, mg/dL**	105.5 (36.8)	108.5 (40.0)	3.1 (11.4)	0.32	n/a	n/a	n/a	n/a

Bold = *p *< 0.05

^a^For the change from baseline to 3 months, and baseline to 6 months

### Qualitative outcomes

All community-based SMA participants joined the first focus group after the SMA program. The majority reported by survey that they did not have a hard time following the nutritional recommendations, incorporating the lifestyle changes, taking the supplements, and/or eating the food delivery meals of the SMA program. Themes were grouped into three categories: (1) education and health changes due to the SMA program; (2) program preferences; and, (3) thoughts about future state.

#### Education and health changes

Participants shared several health changes as a result of the community-based SMA program. These included a greater understanding of food as medicine and inflammatory foods, more water intake, improved gut function, more mindful eating practices, and improved energy levels. Participants discussed the connection between program activities and function:

*My thing is that it allows me to function, and function better. By eating the right foods, taking supplements, being mindful of what and when I eat. (Female participant)*

*Eat slowly. I ate so fast, they taught me to place fork down, and chew food thoroughly never used to chew, my food, and I’m enjoying the flavors in the food. (Female participant)*

*The routines help your body to feel better, more energy, I started jogging.* (*Female participant)*

#### Program preferences

Several factors influenced the uptake of the community-based SMA program recommendations. Participants appreciated the time for and method of explanation given for nutrition choices, test results, and use of dietary supplements. A few commented that this was different than their current experience with medical care.

*Most places don’t explain why you shouldn’t eat something, just to not eat it. (Female)*

*I liked the cardio metabolic testing they did. If you were to go to your doctor, they wouldn’t do that… [The SMA program staff] tell you what to focus on. No doctor has every mentioned that. (Female)*

Community-based SMA participants appreciated the in-kind resources of meal delivery, dietary supplements, and laboratory testing. They especially liked the curriculum and delivery of the program, including the cooking demonstration session. The value of the group support was also discussed.

*I like the way you focus on how’s and whys, why you do something. The group support was a main reason. We supported each other on things. (Female)*

Participants’ preferences for changes to the program included more variety in food choices, even more individualized support, and more cooking demonstrations. Possible additions to the program included adding a physical activity component and ensuring an affordable program if supplements and food were not covered.

#### Future state

SMA participants had two main thoughts on next steps. The first was that they had received enough information to be in control of their health journey. The second was concern about continuing the habits without the group and the meal delivery/food component due to time and budget constraints. In response to the concerns, the community engagement team provided health activities to facilitate participants meeting and being accountable to their goals.

*In learning what foods to eat to help blood pressure and cholesterol, we knew this, but being in group helped so much. I have been on millions of diets, and this is the first time I was able to lose weight, lower blood pressure, and cut my high blood pressure medicine. And now, I know where to go. (Female)*

*The program is not a piece of cake, you have to get off the couch. We have done healthy challenges, so this part wasn’t too difficult for us. But, with diet, it’s very hard because you have to pass a bunch of fast food restaurants when you’re getting from place to place, think about grabbing this grabbing that. You have to work at diet to stick with it. (Male)*

A second focus group with eight participants from the community-based SMA program was conducted at 6 months (i.e., 3 months after the completion of the program). All maintained several aspects of the program, including increased healthful food and water consumption. Barriers to maintenance stemmed from the loss of weekly sessions and the loss of prepared food (meal delivery) to support portion size and food choices.

*Well one thing about being by myself, is that I don’t cook that much. Like if I do cook something, and I cook a big amount, it is all gone. Those dinners helped me a lot with the portion. Then when I tried to figure out what was too much by using the palm of my hand, I realized I had two pieces of chicken. I really have to work on that. It’s really hard. But the meals were helpful. (Female)*

Facilitators for maintenance included preparing meals in advance, recalling learnings from the SMA program, receiving positive feedback on health, and sharing health education with family and friends.

*I actually got a little lazier after the program because we were receiving the meals and we did not have to cook. It was really time saving. I ended up getting the meals again because I thought that was the easiest for me and the easiest way out. It isn’t difficult though. This Sunday I cooked three different things that would last for the week. Which I could’ve been doing; saving that Sunday to do all the time but it was football season at the time. (Male)*

*When I went to the hairdresser, they told me that my hair got stronger and that there was more hair. (Female)*

*I went to my regular doctor and they told me that whatever I’m doing, I should keep doing. They said I am in excellent shape and to continue doing what I’ve been doing. (Female)*

Table 4 illustrates key themes for implementation as discussed with the community-based SMA program stakeholders. Barriers to sustainability include heavy time and personnel resources to carry out the program and need to adapt the program to community participants with significant medical and socioeconomic needs. Facilitators to implementation included enthusiastic clinical staff who were familiar with the content and could adjust to a lower health literacy group than seen in clinic-based SMAs, partnership with a trusted community organization, and ability to obtain in-kind resources to support participants’ full engagement in the program.

**Table 4:** Stakeholder interviews findings on the implementation of community-based SMA program

Implementation Questions	Themes
What are the organizational resources to carry out the FFL Community program?	• Existing program adapted to community setting • FTE support for multidisciplinary team - dietitian, health coach, clinician, and administrative teams to deliver the program • Ability to obtain in-kind donations for meal delivery, dietary supplements, laboratory testing, and printed health education material • Existing community health activities and team that facilitated the selection of participants
What are the staff experience and capacity to carry out FFL Community?	• Significant clinical staff experience and passion to adapt the SMA program for a group with more health and socioeconomic needs than usual patients who may not have financial or environmental barriers • Cultural competency and authenticity of providers
What are the potential barriers to implementing FFL Community?	• Significant time preparation for those that delivered the program content to meet participants’ health literacy and morbidity level (e.g., most participants did not have computers or wifi access so all materials needed to be printed) • Large group as there was no attrition among the participants • Lack of space in community setting made one-on-one sessions challenging for physical exam or lab review • Lack of staff knowledge about local community resources - unsure if enough tools provide for participants to continue program on their own given environment and health conditions • Sustainability and scaling limited as program was resource intense and non-revenue generating; dependent on philanthropic support • Distrust between community residents and healthcare system
What are the potential facilitators to implementing FFL Community?	• Ongoing communication and engagement between the clinical and community teams for planning, flexible execution, and evaluation • Weekly reminder calls to participants from the community team • Participants were familiar with each other, health activities, and the community site which facilitated group engagement • Meal delivery facilitated nutrition; unclear if sustainable
What potential modification to FFL Community would need to be made to maximize implementation?	• Better understanding of community members’ needs and assets to adapt program content • More experiential learning, such as cooking demonstrations and grocery store shopping on food vouchers • Develop and incorporate activities for longer support of participants

## Discussion

The community-based nutrition and lifestyle-focused SMA program was able to improve various self-reported wellness and biometric outcomes for patients living in a resource-challenged neighborhood. Additionally, patients described health changes in energy, gut function, and overall function as a result of the SMA program, and many were able to sustain healthy behaviors for at least 3 months post-program, despite cessation of meal delivery services. These findings extend recent findings in a clinical-setting [6] and demonstrate that this healthcare delivery option is a viable and acceptable option in underserved communities where there is a disproportionate burden of chronic disease.

Findings reported here are consistent with the few, available studies evaluating SMAs in underserved community-based settings. Briefly, Batsis and colleagues [19] demonstrated that a 16-week, virtual program (mix of in-person and group visits) targeting obesity in a rural population resulted in an average weight loss of approximately 5.0 lbs. which is similar to what was achieved in the current study. Although the population was predominantly white, a rural population does have a higher prevalence of obesity [20] and face similar barriers to healthcare access as underserved populations due to transportation difficulties, distance to facilities and financial limitations [21]. Additionally, while the virtual aspect of their program was reported as beneficial (e.g. less commute time, less expensive than traveling), participants did report that more in-person programming within groups would allow for more communication and support which was a benefit conferred as part of the current study [19].

Emmert-Aronson and colleagues [22] evaluated Open Source Wellness, a 16-week behavioral pharmacy that prescribes physical activity, healthy meals (by way of vouchers), social support, and stress reduction within a federally qualified health center. They demonstrated increased fruit and vegetable consumption and significant reductions in BMI (b = 0.31, *p* < 0.01) and systolic BP (b = -4.04, *p* < 0.01) in a predominantly African American population. While systolic blood pressure improvements did not reach the magnitude of the current study, they report a subset of hypertensive patients achieved nearly 20 point improvements in systolic blood pressure; however, this is unpublished data [23]. More recently, Crane and colleagues demonstrated that a 12-week, group-based intervention delivered within an African American church community significantly improved median weight (-5.7 lb.), HbA1c (− 0.5%), and systolic BP (-13.3 mmHg) [24]. These findings are similar to those achieved in the current program with the exception of HbA1c which did not improve.

Participants appreciated the content delivery, longer duration for patient education, and peer support. This perception resonates with other studies that have found that group visits as a critical component of SMAs can facilitate more equitable patient-provider dynamics than standard care because of the presence of multiple patients and the extended amount of time providers spend with a group of patients which may be especially important for low-income, African American patients who may have distrust of medical settings [25].

Partnership and engagement with trusted community organizations is needed from the outset for nutrition-related community-based, collaborative interventions [26]. The acceptability of the community-based SMA program was in part due to a prior trust between participants and the community engagement staff. The staff were supportive of the SMA program and helped facilitate participant attendance and program implementation. However, sustainability and scaling barriers include significant personnel time and need for philanthropic support (i.e., unknown if cost-effective) to deliver the SMA in low-income settings.

This study has several limitations. First, the overall design was a pre-post comparison study with no control group. Second, there was selection bias for participants chosen by the community engagement team to participate in the SMA program. Specifically, participants were chosen based on their passion for health and wellness after successful completion of other community health programs. Therefore, study participants’ likely had higher self-efficacy that contributed to greater adherence and session attendance than another group of participants. Third, the small sample size and 6 month duration limits the generalizability and sustainability of our findings. Lastly, many outcomes were self-reported, which may be exaggerated due to social desirability reporting bias. Regardless, the current study also included quantitative measures, though comparison p-values may be significant by chance.

## Conclusions

Community-based SMA programs focused on nutrition and lifestyle are acceptable in underserved communities and can lead to improvements in health-related outcomes and behaviors. However, future studies are warranted to evaluate various factors including long-term program sustainability, community participant engagement, and the impact of improved health literacy. Additionally, examining the resource-effectiveness of community-based nutrition and lifestyle-focused SMA programs to underserved communities would be beneficial in order to appreciate scalability.

## Data Availability

The datasets generated and/or analyzed during the current study are not publicly available due confidential personal health information but are available from the corresponding author on reasonable request.

## Abbreviations

SMA: shared medical appointments
HbA1c: Hemoglobin A1c
LDL: Low-density lipoprotein
